# Active Pharmaceutical Ingredient Uptake by Zebrafish (*Danio rerio*) Oct2 (slc22a2) Transporter Expressed in *Xenopus laevis* Oocytes

**DOI:** 10.1002/etc.5480

**Published:** 2022-10-12

**Authors:** Elisabeth D. Chang, Stewart F. Owen, Christer Hogstrand, Nic R. Bury

**Affiliations:** ^1^ Division of Diabetes and Nutritional Sciences King's College London London UK; ^2^ AstraZeneca Macclesfield Cheshire UK; ^3^ School of Ocean and Earth Science University of Southampton Southampton UK

**Keywords:** Absorption, emerging pollutants, in vitro toxicology, pharmaceuticals

## Abstract

Uptake of active pharmaceutical ingredients (APIs) across the gill epithelium of fish is via either a passive or facilitated transport process, with the latter being more important at the lower concentrations more readily observed in the environment. The solute carrier (SLC) 22A family, which includes the organic cation transporter OCT2 (SLC22A2), has been shown in mammals to transport several endogenous chemicals and APIs. Zebrafish *oct2* was expressed in *Xenopus* oocytes and the uptake of ranitidine, propranolol, and tetraethylammonium characterized. Uptake of ranitidine and propranolol was time‐ and concentration‐dependent with a *k*_m_ and *V*_max_ for ranitidine of 246 µM and 45 pmol/(oocyte × min) and for propranolol of 409 µM and 190 pmol/(oocyte × min), respectively. Uptake of tetraethylammonium (TEA) was inhibited by propranolol, amantadine, and cimetidine, known to be human OCT2 substrates, but not quinidine or ranitidine. At external media pH 7 and 8 propranolol uptake was 100‐fold greater than at pH 6; pH did not affect ranitidine or TEA uptake. It is likely that cation uptake is driven by the electrochemical gradient across the oocyte. Uptake kinetics parameters, such as those derived in the present study, coupled with knowledge of transporter localization and abundance and API metabolism, can help derive pharmacokinetic models. *Environ Toxicol Chem* 2022;41:2993–2998. © 2022 The Authors. *Environmental Toxicology and Chemistry* published by Wiley Periodicals LLC on behalf of SETAC.

## INTRODUCTION

Carrier‐mediated cellular transport plays a critical role in the uptake of hydrophilic active pharmaceutical ingredients (APIs) across plasma membranes in mammals (Koepsell, 2020, 2021). This drug transport is mediated by organic cation transporters (OCTs) that include members of the solute carrier proteins (SLC) 22A (e.g., OCT1 [SLC22A1]; OCT2 [SLC22A2]; OCT3 [SLC22A3]; OCTN1 [SLC22A4]; OCTN2 [SLC22A5]; PMAT [SLC29A4]), and the multidrug and toxic compound extrusion (MATE) family (MATE1 [SLC47A1]; MATE2 [SLC47A2]; Koepsell, 2021). Many of these transport proteins are described as being polyspecific, that is, they can interact with compounds of various molecular structures and sizes, thus exhibiting large differences in substrate turnover and affinity that can transfer several endogenous compounds, xenobiotics, and metabolites in addition to drugs (Koepsell, 2021). A wide range of chemical substrates have been shown for the zebrafish slc22a6 and 8 (Dragojević et al., 2019, 2020), slc22a1 (Mihaljević et al., 2017), and members of the slc47 family (Lončar & Smital, 2018; Lončar et al., 2016) when expressed in mammalian cell lines. These transport proteins play a key role in chemical and drug absorption, distribution, metabolism, and excretion (ADME) processes (Koepsell, 2020, 2021).

The OCT2 protein‐facilitated transport is sodium‐independent and relies on an electrochemical gradient for cation transport (Arndt et al., 2001; Busch et al., 1998; Volk et al., 2003). Mammalian OCT2 proteins are typically expressed on the basolateral membrane of the proximal tubules of the kidney where OCT2 transports organic cations from the blood into the cells. Clearance from the cells into the tubular filtrate is then facilitated by MATE proteins and thus both are essential for organic cation homeostasis. The substrate specificity of OCT2 overlaps with OCT1 and OCT3 but exhibits substantial differences in substrate affinity and can transport compounds including neurotransmitters (e.g., histamine, serotonin), endogenous compounds (e.g., guanidine, choline), model compounds (e.g., triethylammonium), and various drugs (e.g., amantadine, cimetidine, famotidine; Volk et al., 2003). In zebrafish, the expression profile analysis of *oct2* transcript shows a wide tissue distribution: in addition to the kidney the transporter is found in the eye, testis, and spleen (Mihaljević et al., 2016), as well as the intestine, liver, and gill (Mihaljević et al., 2016), but the substrate specificity of this transporter has yet to be determined.

Previously, we have demonstrated uptake of pharmaceuticals across the gills of fish using a primary fish (rainbow trout) gill cell culture, and identified both passive and facilitated uptake process, with passive uptake dominating at higher concentrations (>100 µg L^−1^) and facilitated at lower environmentally relevant concentrations (<0.14 µg L^−1^; Chang et al., 2021a; Stott et al., 2015). In this in vitro system propranolol uptake was significantly inhibited by quinidine and cimetidine, both known substrates for mammalian OCT2 (Volk et al., 2003), and influenced by external water pH (Chang et al., 2021a; Stott et al., 2015). Thus, we hypothesize that fish Oct2 may facilitate propranolol and other drug uptake across the gills in fish. The aim of the present study was to assess the uptake of tetraethylammonium (TEA) and ranitidine (a histamine H2‐receptor antagonist), both substrates for mammalian OCT2 (Koepsell, 2021; Sandoval et al., 2018; Volk et al., 2003), and the beta‐blocker propranolol by a zebrafish Oct2. To isolate this transporter for uptake studies we used the *Xenopus* oocyte expression system model that has been used extensively to assess protein substrate uptake (Goldin, 1993).

## MATERIAL AND METHODS

### Synthesis of cRNA

The complete *Danio rerio slc22a2* (zgc:64076; IRBOp991A024D; *oct2*) cDNA clone was obtained from Source BioScience (current availability from GenScript® clone ID ODa31637) and inserted into the in a pCMV‐SPORT 6.1 vector (Addgene). The pCMV6‐slc22a2 plasmid was linearized and then purified by addition of two volumes of 100% ethanol, incubation at −20 °C for 15 min, centrifugation at 14 000 *g* for 15 min, removal of supernatant, and resuspension in nuclease‐free water. cRNA was made from linearized plasmid using a SP6 mMessage mMachine kit (Ambion) following the manufacturer's protocol. Concentration and quality were measured using a NanoDrop‐1000 and a sample run on a 2100 Bioanalyzer to check the size of the amplicon.

### *Xenopus laevis* transporter expression

*Xenopus laevis* oocytes were purchased from the University of Portsmouth and prepared on the day of their arrival. The oocytes were washed with calcium free OR‐2 (mM: 90 NaCl, 1 KCl, 5 HEPES, 2.5 pyruvate, 0.06, penicillin, 0.03 streptomycin, adjusted to pH 7.4 with NaOH) and then separated from their membranous sacs mechanically (with forceps) in a Petri dish. Following this, the oocytes were treated with collagenase (1.3 IU ml^−1^) in calcium‐free OR‐2 and incubated at room temperature for 30 min in the dark. After the 30‐min incubation, the collagenase was replaced with calcium‐free OR‐2 media and the oocytes underwent further mechanical separation. Following this, a second collagenase incubation was conducted for a further 30 min. After the collagenase media was removed, the oocytes were washed with OR‐2 media containing 2 mM CaCl_2_. Finally, oocytes were isolated and transferred into six‐well plates containing OR‐2 media and kept at 19 °C overnight.

Oocytes were injected using a Nanoject II auto‐nanoliter injector (3‐000‐204; Drummond). The needles used were made by pulling replacement capillaries with a Sutter P‐87 flaming brown micropipette puller (machine settings: heat 749, pull 63, velocity 45, pressure 300, time 210). Pipettes were loaded with 4–8 μl of *oct2* cRNA or water to act as an injection control, and the remainder loaded with ≈10 μl of mineral oil. The needles were loaded onto the Nanoject and ≈50 nl/oocyte injected with a foot pedal into oocytes arranged on a plastic grid fixed to a Petri dish containing OR‐2.

### Transport assays

Transport assays were conducted 3 days post‐injection of the oocytes and incubation period that allowed for the greatest expression of the transporter. First, 10–20 cRNA‐injected or sham (water‐injected) oocytes were transferred into tubes and washed once with OR‐2 media. The media was then aspirated from the tube and 970 µl of OR‐2 media containing radioactive ^3^H‐ranitidine (2.5 Ci mmol^−1^; Moravek Biochemicals), ^3^H‐propranolol hydrochloride (29.0 Ci mmol^−1^; Amersham Biosciences), or ^3^H‐tetraethylammonium (TEA; 1.5 Ci mmol^−1^; Amersham Bioscience) and where necessary this was adjusted to the desired concentration with nonradiolabelled compound. At the start three 10‐µl samples were taken from this incubation media for radioactivity counts and determination of the specific activity.

Following the appropriate incubation period, the media was aspirated and oocytes were washed with 3 ml of ice‐cold PBS five times. Individual oocytes were transferred to scintillation vials and incubated for 30 min with 500 µl of 10% w/v sodium dodecyl sulfate, 3 ml of scintillation fluid was added to each vial, and the vials were placed in racks to be counted. This procedure was repeated with control oocytes. Radioactivity was counted using a Beckman Coulter LS6500 Multipurpose Scintillation Counter.

For the time‐course assay *oct2* cRNA was injected and control oocytes were incubated with 200 µM of ranitidine, propranolol, or TEA for 30 s, 1, 3, 5, and 10 min. From this analysis all further transport assays were conducted for 45 s; a time period chosen because it is during the exponential component of the temporal uptake curve and is the optimal period to delineate between the sham and *oct2* cRNA injected oocytes. To conduct concentration assays, the OR‐2 media was prepared with a constant concentration of labelled substrate (as previously described) and various concentrations of unlabelled substrate: 50, 100, 200, and 500 µM. Both *oct2* cRNA injected and control oocytes were incubated in each media for 45 s. For pH assays aliquots of OR‐2 transport media were adjusting by manipulating the pH to produce solutions with a final pH of 6, 7, and 8. This was achieved by addition of 1 M HCl and 1 M NaOH. The transport media was then prepared in scintillation vials as previously described. For the inhibition assays oocytes were incubated in 500 µl of preincubation media for 15 min with the addition of 2 mM amantadine, quinidine, cimetidine, ranitidine, or propranolol. Following this, the media was removed and replaced with OR‐2 transport media containing H^3^ TEA along with the inhibitor of interest.

### Data analysis

The oocyte compound concentrations were converted to picomole per oocyte using the media‐specific activity. GraphPad Prism 8 was utilized for graphical representation. The difference in uptake at each time point or pH was determined via unpaired two‐tailed *t*‐tests and comparisons between the uptake in the presence of each drug inhibitor was assessed via an analysis of variance with post hoc Tukey's tests all conducted using GraphPad Sigma with *p* < 0.05 considered significantly different. Derivation of *K*_m_ and *V*_max_ from the dose‐dependent uptake was assessed and derived from Michaelis–Menten nonlinear fit model in GraphPad Prism 8.

## RESULTS AND DISCUSSION

**Figure 1:**
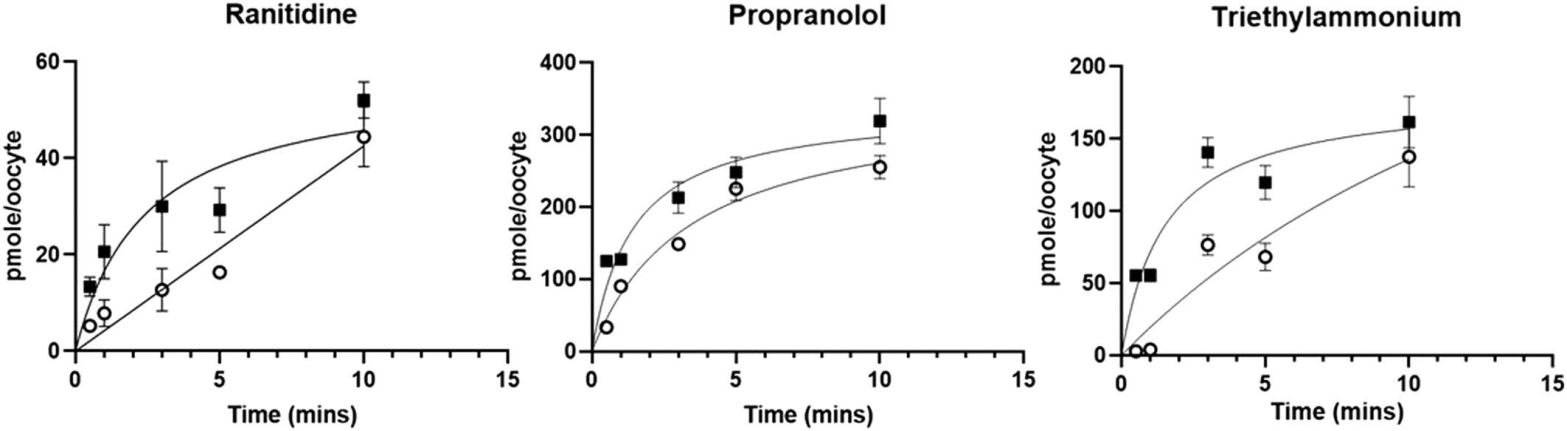
Time‐dependent accumulation of radiolabelled ranitidine, propranolol, and tetraethylammonium into *Xenopus* oocytes expressing zebrafish OCT2 (black squares) or sham injected controls (white circles). Values represent the average ± SEM of between seven and 15 oocytes.

**Figure 2:**
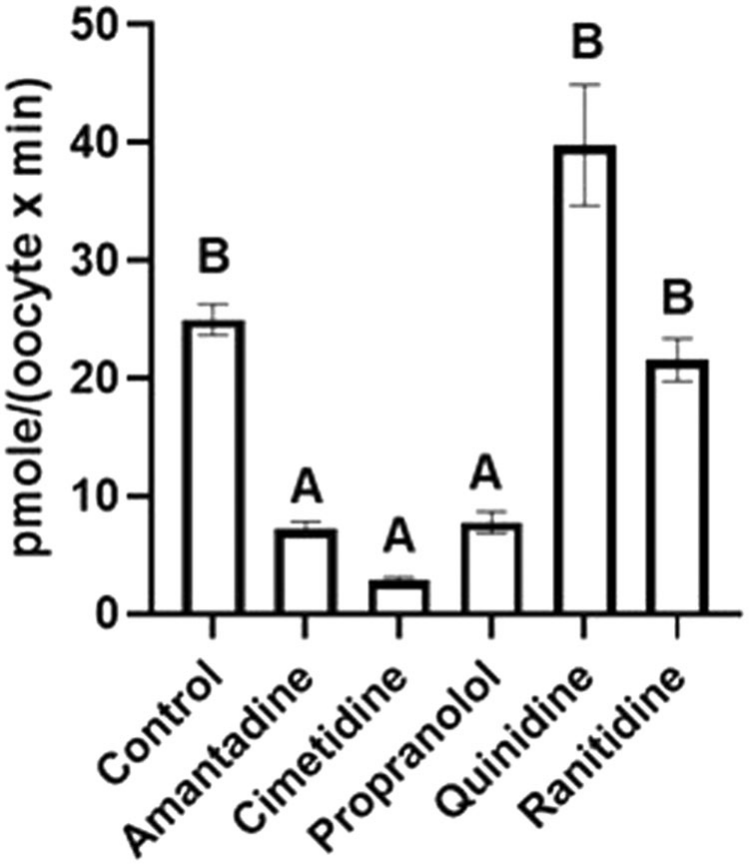
The uptake rate of 200 µM radiolabelled tetraethylammonium into *Xenopus* oocytes expressing zebrafish OCT2 in the absence (control) and presence of 2 mM amantadine, cimetidine, propranolol, quinidine, and ranitidine. Values represent the average ± SEM of between seven and 15 oocytes. The columns with the same letter are not significantly different from each other (one‐way analysis of variance followed by post hoc Tukey's test, *p* < 0.05).

**Figure 3:**
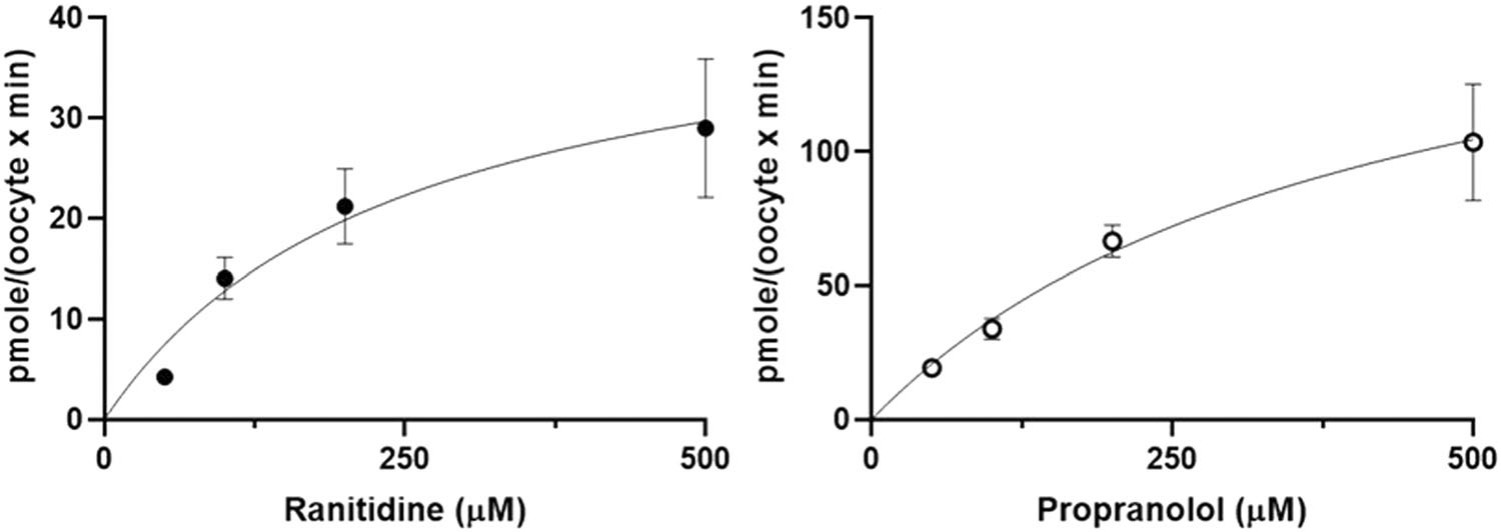
Dose‐dependent uptake rate of radiolabelled ranitidine and propranolol into *Xenopus* oocytes expressing zebrafish OCT2. Values represent the average ± SEM of uptake in seven to 15 OCT2 injected oocytes adjusted for the uptake in sham injected oocytes at each concentration. Michaelis–Menten parameters: ranitidine *k*_m_ = 246.1 µM, *V*_max_ = 44.6 pmol/(oocyte × min); propranolol *k*_m_ = 408.9 µM, *V*_max_ = 190 pmol/(oocyte × min).

**Figure 4:**
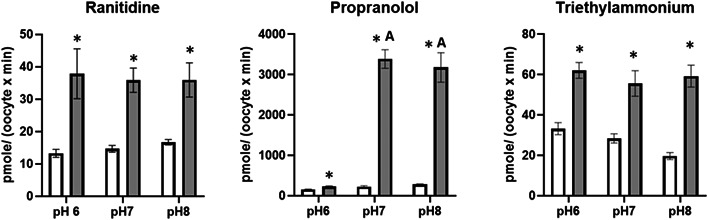
The uptake rate of radiolabelled ranitidine, propranolol, and tetraethylammonium into *Xenopus* oocytes expressing zebrafish Oct2 (grey bars) or sham injected controls (white bars) at pH 6, 7, and 8. Values represent the average ± SEM of between seven and 15 oocytes. Asterisks indicate a significant difference between sham and Oct2 injected oocytes (Student's *t*‐test, *p* < 0.05) and A above the columns indicates a difference in the uptake rate in the Oct2 injected oocytes for propranolol at pH 7 and 8 compared with pH 6 (one‐way analysis of variance post hoc Tukey's test, *p* < 0.05).

The present study shows that zebrafish *oct2* when expressed in *Xenopus* oocytes can facilitate the transport of ranitidine, propranolol, and TEA (Figures 1, 2, 3, 4). This contributes to the knowledge that shows several other zebrafish SLC proteins can transport a wide range of endogenous compounds, including steroids and steroid conjugates, xenobiotic, drugs and their metabolites (Dragojević et al., 2018, 2019, 2020, 2021; Lončar et al., 2016; Mihaljević et al., 2017; Popovic et al., 2013), and are likely to play a significant role in ADME processes in fish.

The observation that propranolol uptake occurs via Oct2 (Figures 1 and 3) supports our previous hypothesis that uptake of this drug from the aquatic environment at environmentally relevant concentrations may be via a facilitated uptake process (Stott et al., 2015), and importantly points to the wider importance of a role for active uptake of environmental pollutants in fish and other aquatic animals. In addition, the transport of TEA by zebrafish Oct2 was inhibited by amanitidine and cimetidine (Figure 2), both known substrates for mammalian OCT2 (Sandoval et al., 2018; Koepsell, 2021; Volk et al., 2003), and these drugs have also been shown to inhibit propranolol uptake via the fish gills (Stott et al., 2015). In addition, both propranolol and ranitidine are substrates for human OCT2 (see references in Koepsell, 2021; Volk et al., 2003). However, the dose‐dependent uptake provides the affinity and maximum transport capacity of zebrafish Oct2 for rantidine (*k*_m_ = 246 µM, *V*_max_ = 45 pmole/[oocyte × min]) and propranolol (*k*_m_ = 409 µM, *V*_max_ = 190 pmol/[oocyte × min]; Figure 3). This affinity for the transporter is higher than that reported for the human OCT2–ranitidine (*k*_m_ = 65 µM) and propranolol (*k*_m_ = 12 µM; Koepsell, 2020), and is orders of magnitude higher than that measured in the environment, picomolar to nanomolar (Miller et al., 2019a, 2021). However, this environmental concentration may not reflect that present at the apical surface of the gill due to entrapment of the drug within the gill microclimate.

The OR‐2 media pH greatly affected the uptake rate of propranolol, but not ranitidine or TEA (Figure 4). Ranitidine has a p*K*a of 8.2 (Kortejärvi et al., 2005) and thus there is large variation of the un‐ionized to ionized ratio at each pH (pH 6, 0.8:99.8; pH 7, 7.7:92.7; pH 8, 45.4:54.6) and the internal ratio of 18.3:81.7. The lack of difference in the observed uptake rates at the different pH values was surprising. Ranitidine's low lipophilicity (Coruzzi et al., 1996) would suggest poor passive membrane permeability, and an increased uptake in oocytes expressing zebrafish *oct2* was initially observed (Figures 1 and 4). In contrast to the present study, an increase in ranitidine uptake was observed because pH increased in Caco‐2 cells (Bourdet & Thakker, 2006). Even though ranitidine is a substrate for human OCT2 (Koepsell, 2020), it has greater transport affinity for human OCT1 when expressed in *Xenopus* oocytes (Bourdet et al., 2005). The lower affinity of ranitidine to OCT2 may account for the inability of ranitidine to inhibit TEA uptake by Oct2, but that hypothesis should be addressed by future studies (Figure 2).

The uptake of propranolol, with a p*K*a value of 9.42 (Avdeef et al., 1998; Fuguet et al., 2008; Pauletti & Wunderli‐Allenspach, 1994), was significantly increased (100‐fold) where the fraction of un‐ionized drug is greatest, albeit that at pH 7 the fraction is 0.8% and at pH 8 it is 3.8%. These observations corroborate previous studies that have shown that minor changes in un‐ionized to ionized ratios have a significant effect on propranolol uptake across a fish gill epithelium model (Chang et al., 2021a; Stott et al., 2015, 2021b). The pH‐dependent uptake of propranolol has also been observed in other epithelia, including retina (Kubo et al., 2013), Caco‐2 cells (Wang et al., 2010), and renal LLC‐PK1 (Dudley et al., 2000). In the present study, the ratio of external to internal concentration of the un‐ionized fraction of propranolol at pH 8 (based on an oocyte volume of 1 µL; Ferrell & Machleder, 1998) is 0.31 and the ionized fraction is 0.07 (Table 1). This suggests uptake of either form is against a concentration gradient. Even though human OCT2 has been reported to be able to transport cations and noncharged compounds, and potentially in both directions across the plasma membrane (Koepsell, 2020), the resting potential of *Xenopus* oocytes is between −40 and 72.5 mV (Corbin‑Leftwich et al., 2018; Dascal et al., 1984) and favors cation uptake.

**Table 1:** Ratio of external (µM_ext_) and internal (µM_int_) concentrations of un‐ionized and ionized ranitidine, propranolol, and tetraethylammonium (TEA) at different pHs

	Ranitidine		Propranolol		TEA	
	(µM_ext_:µM_int_)		(µM_ext_:µM_int_)		(µM_ext_:µM_int_)	
	Un‐ionized	Ionized	Un‐ionized	Ionized	Un‐ionized	Ionized
pH 6	1.6:4.94	198.4:30.2	0:1.7	200:224	0:0	200:62
pH 7	7.7:4.94	192.3:30	0.8:25.39	199.2:3352	0:0	200:55
pH 8	90.8:4.94	109.2:30.2	7.4:23.82	192.6:2584	0.4:0	199.6:59

Tetraethylammonium has a p*K*a of 10.8 (Zucker, 1981) and thus at *Xenopus* oocyte internal pH 7.43 (Ferrell & Machleder, 1998) and pH 6 and 7 TEA will be 100% and only 0.2% is un‐ionized at pH 8, suggesting that for this compound uptake across Oct2 would be via the ionized form. Tetraethylammonium uptake is inhibited by propranolol (Figure 2) and internal un‐ionized concentrations exceed that of the external media (Table 1), thus generating a concentration gradient favoring un‐ionized export.

Pharmacokinetic (PK) models aim to predict the ADME of drugs and other xenobiotics to identify the potential health risks posed to an organism (Wang et al., 2022). Exposure of organisms to chemicals can be used to derive the parameters necessary for these models, such as uptake rate, temporal tissue distribution, abundance and compartmentalization, metabolism, and excretion rates. However, the cost of whole‐animal studies, the time they take, and the ethical issues of using animals has seen the advent of new approach methods (NAMs) that use in vitro, in silico, and in chemico information to replace whole‐animal testing. Pharmacokinetics is a particular challenge for NAMs due to the complexity of physiological systems throughout the animal kingdom. In vitro functional data, such as that derived in the present study, and those for a plethora of  SLC transport proteins in zebrafish (Dragojević et al., 2018, 2019, 2020, 2021; Lončar & Smital, 2018; Lončar et al., 2016; Mihaljević et al., 2017; Popovic et al., 2013), when combined with plasma concentrations, tissue expression and localization data can be used to generate physiologically based PK models for internal distribution. However, for aquatic organisms an important component of bioaccumulation studies is uptake from the water. Derivation of bioconcentration factors is often performed at water concentrations that far exceed those measured in the environment (Miller et al., 2019b). Stott et al. (2015) provided evidence of both passive and facilitated uptake processes of APIs across the gill, with facilitated processes more dominant at environmentally relevant concentrations. The present study provides a line of evidence for the role of Oct2 in the uptake of propranolol and ranitidine in fish.

## Supporting information

The Supporting Information is available on the Wiley Online Library at https://doi.org/10.1002/etc.5480.

## Conflict of Interest

Elisabeth D. Chang, Christer Hogstrand, and Nic R. Bury declare no conflict of interest.

## Disclaimer

Stewart F. Owen is an employee of AstraZeneca, a biopharmaceutical company specializing in the discovery, development, manufacturing, and marketing of prescription medicines, including some products reported in the present study.

## Author Contributions Statement

**Elisabeth D. Chang**: Conceptualization; Methodology; Data curation; Writing—review & editing. **Stewart F. Owen**: Conceptualization; Writing—review & editing. **Christer Hogstrand**: Conceptualization; Funding acquisition; Supervision; Writing—review & editing. **Nic R. Bury**: Conceptualization; Data curation; Formal analysis; Methodology; Project administration; Resources; Supervision; Visualization; Writing—original draft.

## Supporting information

This article includes online‐only Supporting Information.

Supporting information.

## Data Availability

Data, associated metadata, and calculation tools are available from the corresponding author (n.r.bury@soton.ac.uk) and data are uploaded as Supporting Information.
